# HIV-1 Infected Lymphoid Organs Upregulate Expression and Release of the Cleaved Form of uPAR That Modulates Chemotaxis and Virus Expression

**DOI:** 10.1371/journal.pone.0070606

**Published:** 2013-07-29

**Authors:** Manuela Nebuloni, Lidia Zawada, Angelita Ferri, Antonella Tosoni, Pietro Zerbi, Massimo Resnati, Guido Poli, Luca Genovese, Massimo Alfano

**Affiliations:** 1 Pathology Unit, “Luigi Sacco” Department of Biomedical and Clinical Sciences, University of Milan, Milan, Italy; 2 Molecular Genetic Unit, Division of Genetics and Cell Biology, San Raffaele Scientific Institute, Milan, Italy; 3 Vita-Salute San Raffaele University, School of Medicine, Milan, Italy; 4 AIDS Immunopathogenesis Unit, Division of Immunology, Transplantation and Infectious Diseases, San Raffaele Scientific Institute, Milan, Italy; Rush University, United States of America

## Abstract

Cell-associated receptor for urokinase plasminogen activator (uPAR) is released as both full-length soluble uPAR (suPAR) and cleaved (c-suPAR) form that maintain ability to bind to integrins and other receptors, thus triggering and modulating cell signaling responses. Concerning HIV-1 infection, plasma levels of suPAR have been correlated with the severity of disease, levels of immune activation and ineffective immune recovery also in individuals receiving combination anti-retroviral therapy (cART). However, it is unknown whether and which suPAR forms might contribute to HIV-1 induced pathogenesis and to the related state of immune activation. In this regard, lymphoid organs represent an import site of chronic immune activation and virus persistence even in individuals receiving cART. Lymphoid organs of HIV-1^+^ individuals showed an enhanced number of follicular dendritic cells, macrophages and endothelial cells expressing the cell-associated uPAR in comparison to those of uninfected individuals. In order to investigate the potential role of suPAR forms in HIV-1 infection of secondary lymphoid organs, tonsil histocultures were established from HIV-1 seronegative individuals and infected *ex vivo* with CCR5- and CXCR4-dependent HIV-1 strains. The levels of suPAR and c-suPAR were significantly increased in HIV-infected tonsil histocultures supernatants in comparison to autologous uninfected histocultures. Supernatants from infected and uninfected cultures before and after immunodepletion of suPAR forms were incubated with the chronically infected promonocytic U1 cell line characterized by a state of proviral latency in unstimulated conditions. In the contest of HIV-conditioned supernatants we established that c-suPAR, but not suPAR, inhibited chemotaxis and induced virus expression in U1 cells. In conclusion, lymphoid organs are an important site of production and release of both suPAR and c-suPAR, this latter form being endowed with the capacity of inhibiting chemotaxis and inducing HIV-1 expression.

## Introduction

Immunological hallmarks of HIV-1 infection are the progressive depletion of CD4^+^ T lymphocytes cells, immune dysfunction and chronic cell activation and inflammation [1], [2], even in virologically suppressed patients receiving combination antiretroviral therapy (cART). Chronic inflammation is a major driver of co-morbidities and, indeed, HIV-1 infected persons under therapy still have a shorter life expectancy and are at higher risk to develop non-infectious diseases than age-matched uninfected persons [3], [4]. Inflammation and immune activation also control virus replication, thereby participating to cell-cell spreading of HIV infection and homeostatic proliferation of the viral reservoir [5].

Crucial events of HIV-1 induced pathogenesis occur in secondary lymphoid organs such as tonsils and lymph nodes (LN), in which both chronic immune activation and viral spreading occur during the clinically silent phase of infection [6], [7], [8], [9], [10], [11]. Moreover, anti-retroviral drugs not always reach effective concentrations in lymphoid organs, which represent important viral reservoirs also in individuals under cART allowing virus propagation at levels believed to be insufficient to select for drug-resistance but sufficient for replenishing the viral reservoir [12], [13], [14], [15].

HIV-1 infection is known to perturb the plasminogen activator (PA) system. In this regard, binding of the urokinase PA (uPA) to uPAR induces activation of uPA, followed by transformation of plasminogen into plasmin [16], a protease that degrades fibrin in D-dimer [17]. Both uPA and plasmin cleave uPAR in the linker region connecting domain I and II, resulting in the presence of cell-associated cleaved uPAR (c-uPAR, composed by domains II and III). Therefore, uPAR might be present at the cell membrane as both full-length and cleaved form (uPAR and c-uPAR, respectively), and the two receptors are also shed as soluble molecules (suPAR and c-suPAR, respectively) by the action of phosphatidylinositol-specific phospholipase D acting at the GPI-anchor shared by both uPAR and c-uPAR [18], [19]. Plasma levels of suPAR, c-suPAR and D-dimer have been correlated with the severity of HIV-1 disease and state of immune activation even in individuals under cART [20], [21], [22], [23], [24], [25], [26], [27]. Of note is the fact that the plasma levels of suPAR and D-dimer in HIV-1^+^ individuals have been shown to represent predictors of disease progression, opportunistic diseases and mortality independently of viremia levels and of CD4^+^ T cells counts and that they were correlated with other inflammatory markers [20], [21], [22], [23], [24], [25], [26], [27]. These observations support the hypothesis that these mediators might play an active role in inflammatory processes and inflammation-driven HIV-related comorbidities. *In vitro*, we and others have previously demonstrated that uPA inhibits virus expression in chronically infected cells and HIV-1 replication in acutely infected primary cells and cell lines [28], [29] in a signaling-dependent manner [28], [30], [31] and that suPAR prevented this effect [32]. However, whether HIV-1 infection affects the uPA/uPAR system in lymphoid organs and whether such a system plays a role in HIV-1 infection in these organs is currently unknown.

To address these questions, tonsils and LN from uninfected and HIV-1^+^ individuals, and tonsils from one HIV-1^+^ patient receiving cART, were investigated for expression of uPA/uPAR components. In addition, some of these tissues were infected *ex vivo*. The kinetics of expression of soluble and tissue-associate uPA, uPAR, c-uPAR, PA inhibitor-1 (PAI-1), CCL2/monocyte chemotactic protein-1 (MCP-1) and HIV-1 replication were evaluated.

In the present study we have also investigated the expression of the chemokine CCL2/MCP-1, because it has been shown to be upregulated by HIV-1 infection both *in vitro* and *in vivo* [33], [34], [35] with an overlapping pattern to that of the uPA/uPAR system [35], [36], [37]. In fact, both CCL2/MCP-1 [38] and suPAR [20] levels have been correlated with HIV-1 disease progression. Furthermore, uPA has been reported to increase CCL2/MCP-1 expression [39] whereas CCL2/MCP-1 was shown to reduce uPA secretion [40]. Finally, c-suPAR was demonstrated to inhibit CCL2/MCP-1 induced chemotaxis [41].

Furthermore, we here report that c-suPAR, but not suPAR, present in the conditioned culture supernatants from *ex-vivo* and *in vivo* HIV infected histocultures modulated the chemotaxis and virus expression in the chronically infected cell line, U1.

Our study provides the first evidence for a distinct role of cell-associated and soluble forms of uPAR in terms of expression in lymphoid organs infected with HIV-1. We also provide evidence for an active role of one of its soluble forms, i.e. c-suPAR, in terms of inhibition of chemotaxis and induction of virus expression. This study bears a relevant translational component in terms of its potential exploitation for the design of novel therapeutic strategies aimed at controlling immune cell activation and HIV-1 reactivation in HIV-infected individuals under cART.

## Materials and Methods

### Analysis of Human Secondary Lymphoid Tissues from HIV-1^+^ and Control Seronegative Individuals

Tonsils and LN from uninfected and therapy-naïve HIV-1^+^ individuals were collected and drawn at the “Luigi Sacco” Hospital, Milan, during a period of 8 years (1994–2001). Nine tonsils (4 from uninfected and 5 from HIV-1^+^ individuals) and 20 LN (12 from uninfected and 8 from HIV-1^+^ individuals) were examined for this study (Table 1). Tonsils from HIV uninfected individuals were used to match the clinical condition present in HIV-1^+^ individuals. All tonsils were from individuals affected by bacterial tonsillitis and were surgically removed after antibiotic therapy. LN were obtained from patients undergoing surgical resection of large bowel because of non-neoplastic disease. Tissues were fixed in 10% buffered formalin and embedded in paraffin. Informed consent was signed and obtained from all participants.

**Table 1:** Clinical parameters of seronegative and HIV-1+individuals used for retrospective analysis.

	HIV-1 Uninfected	HIV-1^+^
**Tonsils**		
Number	4	5
Median age (range)	20 years old (16–24)	21 years old (16–26)
Sex	3 M, 1 F	4 M, 1 F
Histological diagnosis	Follicular hyperplasia (4/4)	Follicular hyperplasia (5/5)
**Lymph nodes**		
Number	12	8
Median age (range)	59 years old (30–75)	42 years old (30–51)
Sex	7 M, 5 F	6 M, 2 F
Histological diagnosis	Follicular hyperplasia (7/12), normal histology (5/12)	Follicular hyperplasia (3/8), normal histology (5/8)

Tonsils with histological diagnosis of follicular hyperplasia from HIV infected and uninfected individuals were analyze.

Lymph nodes with follicular hyperplasia and normal histology from HIV infected and uninfected individuals were use. n; number of individuals, y; years, M; male, F; Female.

Age is report as median and range of distribution in brackets.

### *Ex vivo* Histocultures and HIV-1 Infection of Human Tonsils

Tonsils from 46 HIV-uninfected subjects were collected at the Ospedale San Raffaele, Milan, after receiving formal written waiver from the institutional review board (Ethic Committee, Ospedale San Raffaele) and signed written informed consent from the patients. Tonsillectomy was performed because of frequent annual relapsing tonsillitis; tonsils were surgical removed after antibiotic therapy.

Tonsils were cultivated and infected *ex vivo* according to published protocols [42], [43]. Briefly, 9 tissue blocks were placed on medium-hydrated collagen sponge (Gelfoam; Johnson&Johnson, Skipton, UK) and infected with 5 µl of viral stocks from the reference CCR5-dependent (R5) and CXCR4-dependent (X4) HIV-1 strains BaL and LAI/IIIB, respectively, that were loaded on top of each tissue block. Twenty-seven tissue blocks (subdivided onto 3 collagen sponges) were used for each experimental condition, and used for *in vitro* histoculture, formalin-fixed/paraffin-embedded for histology and immunohistochemistry, snap-frozen in liquid nitrogen for Western blot analysis.

The hyperplastic tonsils from a single HIV-1^+^ individual under cART (male, 26 years old, and cART composed of tenofovir/emtricitabina/efavirenz, with undetectable plasma viremia, <37 copies/ml, at the time of tonsillectomy) were also collected. This case was code as MA35.

### Immunohistochemistry (IHC)

IHC analysis was performed on formalin-fixed/paraffin-embedded tissues, as previously reported [44], [45]. Briefly, sections were mounted on super-frost slides (Bio-Optica, Milan, Italy), de-waxed in xylene, rehydrated in ethanol and pretreated in a microwave oven (5′-two cycles. 780 W, 0.01 M citrate buffer). Then, the slides were incubated for 2 h at room temperature with a rabbit anti-human uPAR antibody (Ab; 6.3 mg/ml, 1∶500 dilution), rabbit anti-human uPA Ab (5.7 mg/ml, 1∶1,000 dilution), mouse anti-human HIV p24 Ab (clone Kal-1, Dako, Denmark, 1∶50 dilution) [45]. The reactions were revealed by Universal HRP-Polymer Biotin-free detection system (MACH4, BioCare Medical, USA) with 3,3-diaminobenzidine free base (DAB) as chromogen. Negative controls were by omission of the primary Ab. Blinded semi-quantitative count of immunostaining was performed by using a 4-point scale. The most highly reactive area was chosen for each sample and immunostaining was quantified by counting positive cells in five 20×fields according to the following rules: 0 (no staining),+(<25% of immunoreactive cells),++(25–75% positive cells),+++(>75% of immunoreactive cells).

Double immunohistochemical reactions were performed with the following Abs: mouse anti-human CD68 monoclonal Ab (mAb; clone KP1; BioCare; 1∶500 dilution), directed against a fixative-resistant epitope on the macrophage-restricted form of the CD68 molecule; mouse anti-human CD20 mAb (clone L26; Dako, 1∶200 dilution) to detect B lymphocytes; mouse anti-human CD3 mAb (clone PS1; BioCare, 1∶100 dilution) to detect T lymphocytes; mouse anti-human CD35 mAb (clone Ber-MAC-DRC, Dako, 1∶200 dilution) to detect follicular dendritic cells (FDC). Specific secondary Abs conjugated with peroxidase and alkaline phosphatases were chosen (MACH2 Double Stain 2; BioCare): the first reaction was developed by using DAB as chromogen (brown staining) and the second reaction by using Vulcan Fast Red (red staining).

### Quantification of HIV-1 Replication in Histoculture Supernatants

Culture supernatants bathing 3 collagen sponges were harvested every 3 days, pooled and clarified by centrifugation and 0.22 µm filter filtration and stored at −80°C. The supernatant was then tested for the presence of HIV-1 by means of Mg^++^-dependent reverse transcriptase (RT) activity, as reported [42].

### Quantification of uPA, suPAR, PAI-1, CCL2/MCP-1, and Lactate Dehydrogenase (LDH) in Histocultures Supernatants

The levels of uPA, suPAR, PAI-1 and CCL2/MCP-1 were detected by ELISA (R&D Systems, MN, USA), and of LDH [46] by using commercial kit (Sigma, MO, USA).

### Western Blot Detection of suPAR forms and of HIV-1 p24 Gag

Both full-length and cleaved uPAR and suPAR forms and of HIV-1 p24 Gag present either at the cell level or in the culture supernatants were identified by Western blotting [47]. Briefly, 27 tissue blocks were lysed in 1 ml of lysis buffer (50 mM Tris HCl pH 7.5, 150 mM NaCl, 0.5% NP-40, 0.5% sodium dehoxycholate and 2 µl of protease inhibitor cocktail by Sigma): tissues were vortex and kept at 4°C on a rotating wheel for 4 h, spun down at 4°C and clarified supernatant collected. Supernatants containing HIV-1 were inactivated with 0.1% NP-40 for 4 h at 4°C on a rotating wheel. Stably transfected HEK-293 cells were used as positive controls for cell-associated and soluble forms of suPAR [47].

For immunoblotting, tonsils lysates (20 µg), HEK-293-uPAR and HEK-293-c-uPAR cell lysates (3 µg) or 20 µl of either cultured tonsils or of HEK-293 transfected cell supernatants were deglycosylated by treating with 4 µl of denaturing solution (0.5% SDS, 10 mM DTT) and boiling for 3 min at 95°C to denaturate uPAR and c-uPAR under mildly reducing conditions. To remove N-linked glycosylation, 8 µl of deglycosylation buffer (PBS containing 2.25% Triton X-100 and 65 mM EDTA) and 0.5 unit of peptide-N-glycosidase F (from *Elizabethkingia miricola*, Sigma) were added to each sample and incubated at 37°C overnight. Immediately prior to SDS-PAGE, samples were add of 4X Laemly buffer (containing 2% ß-mercaptoethanol) and boiled for three min at 95°C.

Samples were loaded in 12% SDS-PAGE under reducing conditions and transferred onto a nitrocellulose membrane. Membranes were blocked for 2 h with 5% nonfat dry milk in TTBS (20 mM Tris–HCl, pH 7.6, 150 mM NaCl, and 0.1% Tween 20) and probed with either M2 anti-uPAR purified polyclonal Ab (1 µg/ml) [47], anti-actin monoclonal Ab, mAb (Sigma) or anti-HIV-1 p24 mAb (Acris Antibodies), followed by appropriated secondary Ab/HRP conjugate (Amersham-GE), and detection by ECL.

### Transfer of Histocultures Conditioned Supernatant and use of Lamivudine (3TC)

Conditioned culture supernatant (CCS) form either uninfected or *ex-vivo* infected (R5 or X4 HIV-1 strain) tonsils was used in the absence or presence of Lamivudine (3TC, Sigma, used at 10 µM). Freshly established tonsil tissue blocks (n = 30) were suspended in 1 ml of clarified conditioned supernatant, with or without 3TC, and 4 h later transferred on top of collagen sponges and cultivated in standard conditions; tissue blocks treated with CCS added of 3TC were cultivated with culture medium containing Lamivudine for the initial 3 days of histoculture.

### Effect of Histocultures Conditioned Supernatants on Chemotaxis and virus Reactivation in Chronically Infected U1 Cells

The chronically infected promonocytic cell line U1 contains 2 copies of integrated HIV-1 provirus per cell and it is characterized by a state of constitutive viral latency that can be reversed by several stimuli [48], [49], [50], [51]. U1 cells were cultivated in RPMI containing 10% heat-inactivated FBS, 1% glutamine/penicillin/streptomycin [52]. The tonsil culture medium (TCM) and the CCS from either uninfected (Nil-c) or HIV-1 infected (R5-c, X4-c) tissue blocks were immunodepleted with either control Ab or polyclonal anti-human uPAR Ab (clone M2, recognizing both suPAR and c-suPAR) [47] before stimulation of U1 cells.

U1 cells (3×10^5^/ml) were cultivated for 4 days with the above conditioned supernatants before quantification of the RT activity content in CCS and of cell-associated HIV proteins by Western blotting [52]. For chemotaxis, U1 cells (10^5^) were suspended in RPMI and placed in the upper part of Boyden chamber (NeuroProbe, MD, USA) and separated from the bottom part by PVP-free 5µm pore polycarbonate membrane, filled with 28 µl of conditioned histocultures supernatants or TCM diluted 1∶50 in RPMI. After 5 h of incubation at 37°C, 5% CO_2_, cells from the bottom chamber were cytospun on superfrost glass-slide, stained with May-Grunwald Giemsa (MERCK) and mounted with Eukitt mounting medium (Kindler GmbH, Freiburg, Germany). Pictures were acquired at 10×magnification with an optical phase contrast microscope, and analyzed by FireCam software (Leica, Deerfield, IL).

### Statistical Analysis

Results are presented both as raw data and as means ± standard error of the mean (SEM), differences considered significant when *p*<0.05. Statistical analyses were performed using the Graph Pad Prism 5 package. Differences in value distribution between experimental conditions (HIV infected vs. Nil) were assessed by the two-tailed paired T test. Two-tailed Wilcoxon signed-rank test was used for comparing fold of induction of paired experimental conditions (median of HIV-1 infected samples vs. median of 1 set for uninfected samples). Two-tailed Mann-Whitney test was used for comparing distributions of unmatched groups, and used for evaluating significances of data from immunohistochemistry. Two-tailed Spearman test was used to test the medians for correlation among variables that were not-normally distributed.

## Results

### PA System Expression in Secondary Lymphoid Organs of Uninfected and HIV-1^+^ Individuals

We here evaluated by immunohistochemistry (IHC) the distribution and cellular expression of uPA and uPAR in lymphoid organ sections of both HIV-1^+^ and control seronegative individuals. The histological evaluation of hematoxylin-eosin slides revealed follicular hyperplasia in 19/29 cases (all 9 tonsils and 10 lymph nodes) and the remaining 10 lymph nodes had normal histology (Table 1). Hyperplastic tonsils and lymph nodes were characterized by tissue enlargement due to the presence of enlarged hyperplastic follicles within germinal centers.

The IHC analysis revealed HIV-1 p24_Gag_^+^ cells in 4/8 hyperplastic lymphoid organs and in 1/5 lymphoid organs with normal histology of infected individuals. As expected [53], HIV-1 p24_Gag_ positivity was associated to FDC, as confirmed by double staining with anti-CD35 mAb (data not shown).

**Figure 1:**
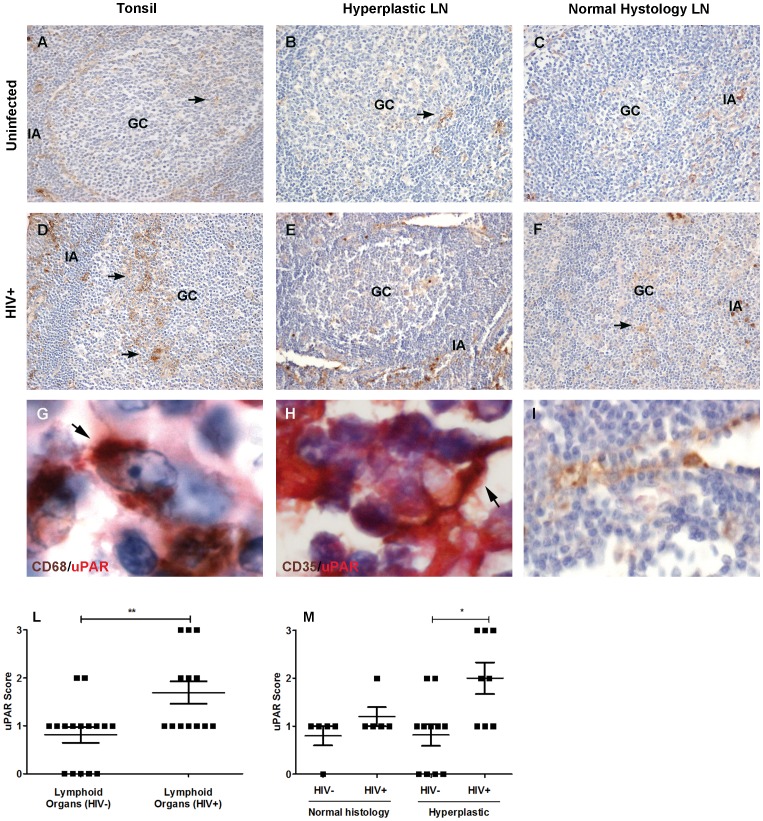
UPAR expression in tonsils and lymph nodes of uninfected and HIV-1^+^ individuals. Figures are representative of tonsils and lymph nodes from uninfected and HIV-1^+^ individuals with hyperplastic and normal histology. Sections used for IHC were obtained from formalin-fixed paraffin-embedded tissues, and the clinical parameters of donors are described in Table 1. Tonsils from either uninfected (panels A-C) or HIV-1+ individuals (panels D-F) show uPAR^+^ cells present both in the GC (arrows, GC) and in the inter-follicular area (IA). UPAR was expressed by macrophages (panel G, double immunohistochemistry with CD68 shown in brown and uPAR in red), FDC (panel H, double immunohistochemistry with CD35 shown in brown and uPAR in red) and endothelial cells (panel I, uPAR shown in brown) in both groups of patients. Panels A-F: Optical Magnification (OM) 10x, panels G and H: OM 100x, panel I: OM 40x. Brown staining in panels G-H is indicated by an arrow. Immunohistochemical score for the number of uPAR^+^ cells was evaluate for lymphoid organs of uninfected and HIV-1^+^ individuals; all lymphoid organs (panel L), normal vs. hyperplastic lymphoid organs (panel M). Vertical and horizontal bars represent mean and SEM. Differences for the number of uPAR^+^ cells in the different tissues and populations were tested by the two-tailed Mann-Whitney test (p values are indicated by asterisk * = 0.01–0.05; **0.001–0.01).

UPAR^+^ cells were found in the germinal centers (GC) and inter-follicular areas of lymphoid organs of both uninfected and HIV-1^+^ subjects (Figure 1A–1F). UPAR was expressed by macrophages and FDC, as confirmed by double immunostaining with anti-CD68 and anti-CD35 mAbs, respectively (Figure 1G, 1H); endothelial cells were also positive for uPAR expression (Figure 1I), whereas T and B lymphocyte were negative for this antigen (data not shown). A semi-quantitative analysis of all samples from lymphoid organs of HIV-1^+^ individuals revealed higher levels of expression of uPAR than what observed in slides of uninfected subjects (Figure 1L). Of interest, 5 out of 16 lymphoid organs from uninfected individuals were negative for uPAR expression, whereas all (13/13) lymphoid organs of HIV-1^+^ individuals were positive (Figure 1L).

Lymphoid organs of both uninfected individuals, either showing normal histology or follicular hyperplasia, and those of HIV-1^+^ individuals displaying normal histology showed a low IHC score for uPAR expression (Figure 1M). A higher IHC score for uPAR expression was observed in hyperplastic lymphoid organs of HIV-1^+^ individuals (Figure 1M). The increased number of uPAR^+^ cells observed in hyperplastic lymphoid organs of HIV-1^+^ individuals was not related to virus expression in that 50% of tissues were negative for p24 Gag. Thus, increased number of uPAR^+^ cells in lymphoid organs of HIV-1^+^ individuals was more likely consequent to virus-induced immune activation, rather than local HIV-1 expression.

**Figure 2:**
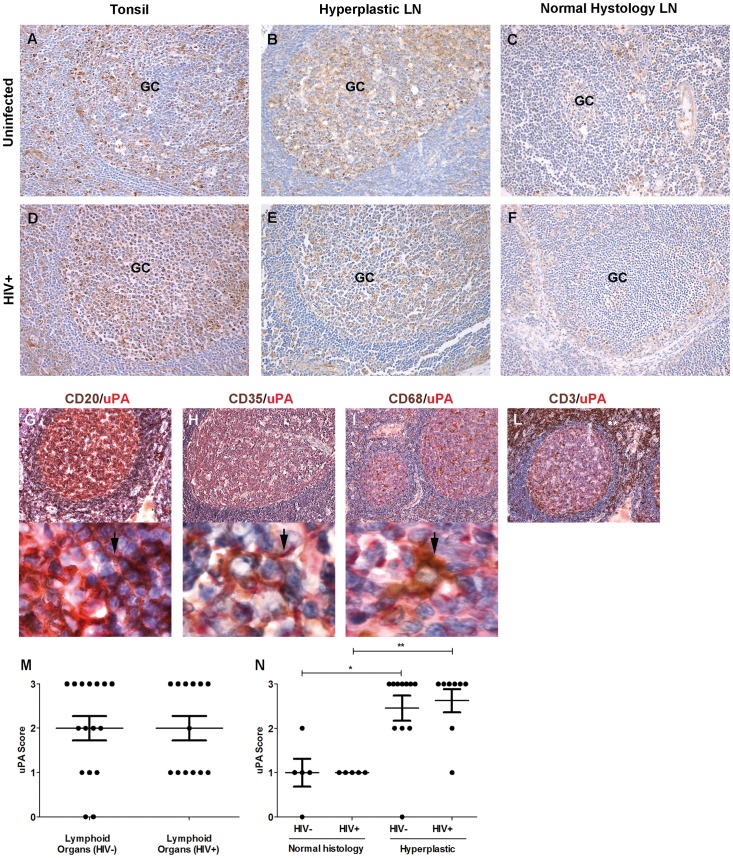
UPA expression in tonsils and lymph nodes of uninfected and HIV-1^+^ individuals. Tonsils from either uninfected (panels A-C) or HIV-1^+^ individuals (panels D-F) showed uPA^+^ cells (in brown) predominantly in the GC. The phenotype of uPA^+^ cells was demonstrated by double immunostaining (panels G-L): uPA (red) was expressed by B lymphocytes (CD20^+^, brown), FDC (CD35^+^, brown) and a few macrophages (CD68^+^, brown), but not T cells (** CD3^+^, brown). Panels G-L: OM 10x. Panels M-O show OM at 100x. Brown staining in panels G-H is indicated by an arrow. Immunohistochemical score for the number of uPA+ cells was evaluate for lymphoid organs of uninfected and HIV-1^+^ individuals; all lymphoid organs (panel M), normal vs. hyperplastic lymphoid organs (panel N). Vertical and horizontal bars represent mean and SEM. Differences for the number of uPA^+^ cells in the different tissues and populations were tested by the two-tailed Mann-Whitney test (p values are indicated by asterisk * = 0.01–0.05; **0.001–0.01).

Conversely, uPA was expressed by B lymphocytes, FDC and some macrophages localized in the GC and inter-follicular areas in lymphoid organs of both uninfected and HIV-1^+^ subjects (Figure 2A–2B vs. 2D–2E). Lower expression of uPA^+^ cells was found in LN with normal histology of uninfected and HIV-1^+^ subjects (Figure 2C vs. 2F), mostly expressed by FDC and some B lymphocytes. The phenotype of uPA^+^ cells was confirmed by double immunostaining (Figure 2G–2I), whereas T lymphocytes (Figure 2L) and endothelial cells (data not shown) were negative for this marker.

A semi-quantitative analysis of all lymphoid organs from HIV-1^+^ patients revealed levels of uPA^+^ cells comparable to those of uninfected subjects (Figure 2M). Considering separately lymphoid organs with normal histology from those with features of hyperplasia (Figure 2N), the IHC score revealed lower expression of uPA in lymphoid organs with normal histology vs. hyperplastic lymphoid organs, irrespectively of the HIV-1 infectious status. Thus, increased number of uPA^+^ cells was typical of lymphoid hyperplasia and was not strictly dependent upon infection.

### Perturbation of CCL2/MCP-1 and of the PA System in Tonsil Histocultures Infected *ex vivo* with HIV-1

In order to investigate whether HIV-1 infection could modulate the PA system in secondary lymphoid organs, tonsil histocultures from HIV-1 seronegative donors were infected *ex vivo* with either R5 or X4 HIV-1 strains. Their culture supernatants were collected and tested for virus replication by means of RT activity, presence of dead cells, and levels of CCL2/MCP-1, uPA, suPAR, and PAI-1. Tissue blocks were analyzed for histology, IHC and Western blotting for expression of uPAR and viral proteins. Results are presented as ratio of the levels of the analytes in infected vs. uninfected tissues (Nil) to minimize inter-donor variability (Figure S1).

**Figure 3:**
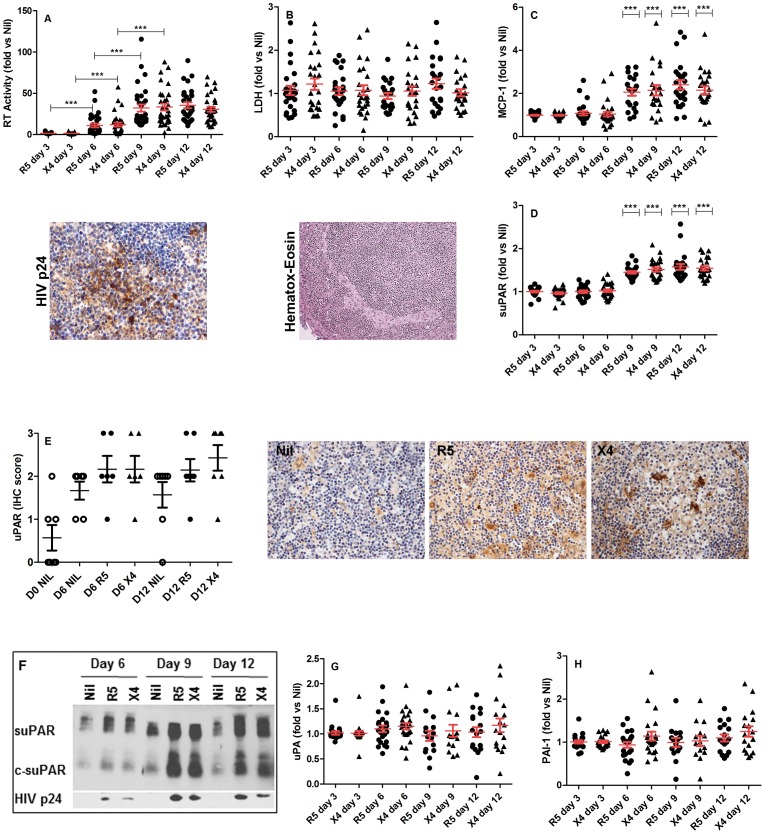
*Ex-vivo* HIV-1 infection of tonsil histocultures increases the soluble levels of CCL2MCP-1, suPAR, c-suPAR and the number of uPAR+ cells. Tonsil blocks were left either uninfected (Nil) or were infected with R5 or X4 HIV-1 strain. Every 3 days the culture conditioned supernatants were tested for the levels of viral replication (RT activity), lactate dehydrogenase (LDH), CCL2/MCP-1, suPAR, uPA and PAI-1. The results are reported as fold vs. Nil (absolute values are shown in the Figure S1). Panels A (n = 31), B (n = 31) and C (n = 28) show fold of HIV-1 replication, LDH and CCL2/MCP-1 levels vs. Nil, respectively, as measured in the culture supernatants The panels below show the IHC detection of HIV p24_Gag_ measured 6 days post-infection and hematoxylin-eosin staining at day 9 after infection. Panel D (n = 28) shows fold of suPAR levels vs. Nil measured in the culture supernatants. Panel E (n = 7) shows the IHC score in terms of number of uPAR^+^ cells in Nil and *ex-vivo* infected tonsil histocultures at the indicated time points after infection (Day 0 is the time of surgical removal); the right panels show the IHC of uPAR+ cells (brown) 9 days post-infection. Panel F shows western blot analysis evaluating for the presence of both uPAR and c-uPAR and of HIV-1 p24 _Gag_ release in the culture supernatants of tonsils left uninfected (Nil) or infected *in vitro* for the indicated time points after infection. Panels G (n = 21) and panel H (n = 21) show the fold of uPA and PAI-1 levels vs. Nil measured in the culture supernatants. The vertical and horizontal bars represent the mean and SEM. Black dots and triangles in panels A-E and G-H represent data from tonsils infected *ex-vivo* with R5 and X4 strain, respectively; white circles in panel E represent data from uninfected ex-vivo tonsils. Statistical significance is indicated by asterisks (*** = <0.001); 2-tailed paired t test (panel A), 2-tailed Wilcoxon signed-rank test (panels C and D). Pictures are at 40×magnification.

Both R5 and X4 HIV-1 replication became evident 6 days after infection (p<0.0001 for R5 and X4 strain at day 6, 9 and 12 vs. Nil defined as 1, n = 31) and achieved a plateau between day 9 and 12 post-infection (Figure 3A); no differences were observed in the replicative capacity of R5 and X4 HIV-1 strains (Figure 3A), as also confirmed by IHC (Figure 3A and Figure S2A). HIV-1 infection did not influence cell mortality in the histocultures, as indicated by the comparable levels of LDH detected in culture supernatants of infected and uninfected tissues (Figure 3B). The histological analysis of both infected and uninfected cultures revealed that the typical structure of tonsils remained evident (presence of GC and of inter-follicular regions), although a decreased cellularity and sharpness of GC was observed at day 9 (Figure 3B) and 12 (not shown) of culture in comparison with earlier time points. Thus, HIV-1 infection *per se* did not influence tissue morphology and viability of tonsil histocultures.

We next searched for the release of CCL2/MCP-1 and elements of the PA system by tonsil histocultures that either were left uninfected or were infected with HIV-1.

CCL2/MCP-1 was detectable in uninfected histoculture supernatants at different times of culture (Figure S1C). As expected, HIV-1 replication increased the secreted levels of CCL2/MCP-1 at day 9 and 12 (Figure 3C). The levels of suPAR were similar in the supernatants of uninfected and infected tissues during the first 6 days of infection, but raised in infected cultures 9 and 12 days post-infection (Figure 3D), in coincidence with virus replication and increased CCL2/MCP-1 secretion.

Immediately after tonsillectomy, no/very few cells were uPAR^+^, but their number expanded during the first 6 days and remained constant in the second week of culture (Figure 3E). Both R5 and X4 HIV-1 infection enhanced the number of uPAR^+^ cells after 6 days of infection and for the following week of culture (Figure 3E). Of note is the fact that the increased levels of suPAR from HIV infected tissue blocks at day 9 and 12 of culture followed the kinetics of virus replication and the increased number of uPAR^+^ cells observed after 6 days of culture.

Next, we evaluated which suPAR forms were released by both infected and uninfected histocultures. Supernatants collected from uninfected cultures contained both suPAR and c-suPAR. Both R5 and X4 HIV-1 infection increased their release as detected 9 and 12 days post-infection (Figure 3F), when virus replication was also detected by either RT activity (Figure 3A) or p24_Gag_ accumulation in culture supernatants (Figure 3F). There was no preferential release of suPAR vs. c-suPAR as also observed in the plasma of HIV-1^+^ individuals [22], [23].

In contrast to these findings, and despite the bi-directional relationship between uPA and CCL2/MCP-1 [39], [40], HIV-1 infection of tonsil did not modulate the levels of soluble uPA and the number of uPA^+^ cells in the same tonsil histocultures (Figure 3G and Figure S2B). However, human tonsils represent a more complex environment than cell lines [39], [40] used to describe the bi-directional relationship between uPA and CCL2/MCP-1, suggesting compensatory mechanisms in lymphoid organs regulating uPA and CCL2/MCP-1 expression upon HIV infection. Moreover, *ex vivo* HIV-1 infection of tonsil histocultures did not modulate the release of soluble levels of PAI-1 (Figure 3H).

### Correlation between suPAR and CCL2/MCP-1 Secretion in Tonsil Histocultures

**Figure 4:**
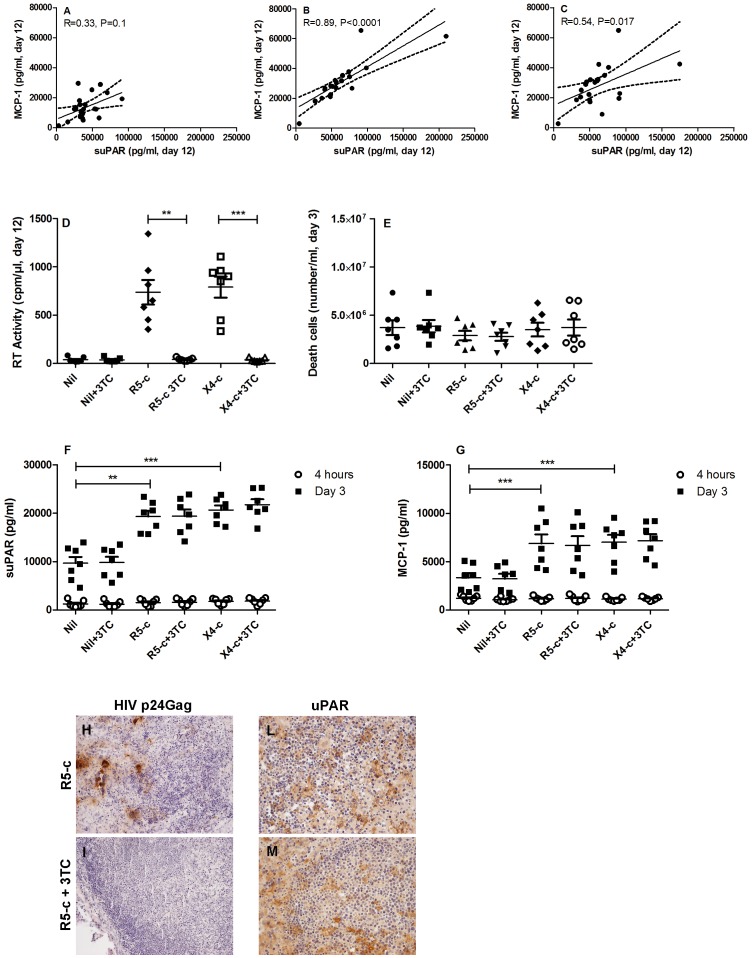
HIV-1 conditioned microenvironment increases the levels of suPAR and CCL2/MCP-1 in uninfected tonsil histocultures. Panels A, B and C show the correlation between the levels of CCL2/MCP-1 and suPAR in the conditioned supernatants of Nil (A) and *ex vivo* infected tonsils (B, R5 strain; C, X4 strain); correlations were analyze by the Spearman test and correlation coefficients (r) and statistical significance (p) are shown within each panel. Dotted curves represent the 95% confidence band of best-fit regression lines. The conditioned supernatants from R5 (R5-c) and X4 (X4-c) infected tissue blocks were used for infecting novel tissue blocks, in the absence or presence of 3TC (10 µM; added during tissue blocks preparation to the conditioned culture supernatant during the 4 h period of incubation, and for the initial 3 days of cultivation of tissue blocks on collagen sponges). Aliquots of culture supernatant were also collected after 4 h of histoculture for measuring the contribution of the “carry over”. Culture supernatants were test for RT activity (D), number of cell death (E), levels of suPAR (F) and CCL2/MCP-1 (G). Two-tailed paired T test (n = 7) was used for statistical analysis, and p values are indicated by asterisks (** = 0.001–0.01; *** = <0.001). Panels H and I show the IHC analysis of HIV-1 p24_Gag_ expression in tonsil histocultures infected with R5-c in the absence and presence of 3TC, respectively. Lack of p24 positivity was observed in 3TC-treated tissue. OM 20x. Panels L and M show the IHC analysis for the expression of uPAR in tonsils infected with R5-c in the absence and presence of 3TC, respectively. No difference of immunostaining was found. OM 20x. Vertical and horizontal bars represent the mean and SEM.

Unlike what observed in uninfected histocultures (Figure 4A), a significant correlation was observed between the secreted levels of suPAR and those of CCL2/MCP-1 in supernatants of both R5 and X4 HIV-1 infected tissue blocks (Figure 4B and 4C), indicating that during HIV-1 infection a common mechanism of regulation or interdependency of the expression of suPAR and CCL2/MCP-1 occurred.

To investigate the potential capacity of HIV-1 replication, or its consequences on the microenvironment, on suPAR and MCP-1 secretion we next investigated whether supernatants from infected histocultures could induce expression of suPAR and CCL2/MCP-1 in the absence or presence of HIV reverse transcriptase inhibitor Lamivudine (3TC). Freshly established tonsil histocultures were incubated with R5 or X4 conditioned supernatants (R5-c and X4-c, respectively). Both supernatants productively infected the new tissue blocks inducing similar levels of virus replication, which was blocked by 3TC (Figure 4D) in the absence of significant changes in terms of cytopathicity in the different experimental conditions (Figure 4E). The levels of both suPAR (Figure 4F) and CCL2/MCP-1 (Figure 4G) released by the new histocultures were not modified by 3 days incubation with 3TC in uninfected conditions. In contrast, both R5-c and X4-c induced higher levels of suPAR and CCL2/MCP-1 vs. uninfected cultures and this effect was not affected by 3TC (i.e. it occurred independently of virus replication, Figure 4F and 4G). In order to unveil the “carry-over” effect of the conditioned supernatants, both suPAR and CCL2/MCP-1 levels were measured after 4 h of culture showing no differences between uninfected tissue blocks and those that had been previously incubated with R5-c and X4-c (Figure 4F and G). These findings were confirmed by IHC, revealing that 3TC-treated tissues were negative for p24 Gag expression (Figure 4H and I) and that the number of uPAR^+^ cells were similar in both 3TC-treated and untreated tissues (Figure 4L and M). According to these findings, the levels of suPAR and of HIV-1 in the culture supernatants were not inter-correlated (n = 28, data not shown), as previously reported *in vivo* [20].

### C-suPAR, but not suPAR, Modulated U1 Cell Chemotaxis and virus Expression Induced by the Conditioned Culture Supernatant (CCS)

As uPAR, suPAR binds uPA and integrins [54]. On the other side c-suPAR binds to formyl peptide receptor (FPR) FPRL1, inducing chemotaxis of FPR^+^ cells [41], [55]. C-suPAR was also shown to induce the heterologous desensitization of chemokine receptors, therefore inhibiting monocyte migration in response to different chemokines, including CCL2/MCP-1 [41], [55]. Because of the different composition of the extracellular milieu from Nil and HIV-infected tonsils (as here shown for suPAR, c-suPAR and CCL2/MCP-1 in the conditioned mediums collected after 9 and 12 days post-infection), we evaluated the contribution of suPAR and c-suPAR to the modulation of biological activities of CCS collected from uninfected and HIV-infected tonsil histocultures. CCS were used as whole or immune-depleted of suPAR forms, by using a polyclonal anti-uPAR Ab (M2 clone) recognizing a domain shared by both suPAR and c-suPAR [47], and eventually reconstituting them with exogenous suPAR or c-suPAR. Immunodepletion completely removed suPAR forms, as evaluated by ELISA, but did not influence CCL2/MCP-1 levels or HIV content, as measured by ELISA or RT activity levels (data not shown), respectively. We next tested these different CCS preparations for their capacity to modulate either chemotaxis or HIV-1 expression in U1 cells, known to constitutively express both FPRL1 and several integrins. In addition, we have previously reported that U1 cells are characterized by secreting ca. 10-fold less suPAR than primary monocytes [32].

**Figure 5:**
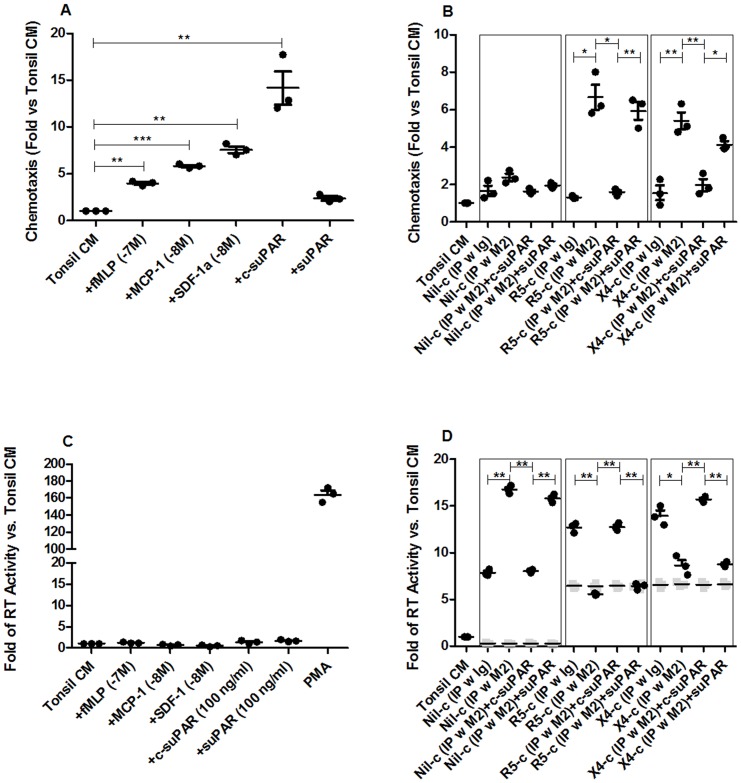
Soluble and cleaved form of uPAR differently modulates chemotactic activity and HIV expression by conditioned microenvironments. Chemotaxis of chronically infected monocytic cells was tested (n = 3) in response to: A. tonsil culture medium (TCM) and TCM added of recombinant stimuli or purified c-suPAR and suPAR [76], [77]; B. conditioned supernatant from uninfected tonsils (Nil-c), R5 infected tonsils (R5-c) and X4 infected tonsils (X4-c) immunodepleted with either control immunoglobulins (IP w Ig) or polyclonal anti-human uPAR Ab (IP w M2) [47] or M2-depleted supernatants reconstituted with 100 ng/ml of purified c-suPAR or suPAR. Migrated cells toward TCM was 25±9 (n = 3). HIV-1 expression from U1 cells was tested (n = 3) by cultivating them with the same stimuli and conditions used and described for chemotaxis assay (panels C-D). Gray squares in panel D represent the value of input virus in the conditioned supernatants at the end of the 4 days of culture. CM from *ex vivo* uninfected and infected tonsils were collected after 9 days of histoculture. Vertical and horizontal bars represent mean and SEM. Two-tailed paired T test was used for statistical analysis, and p values are indicated by asterisks (* = 0.01–0.05; ** = 0.001–0.01; *** = <0.001).

U1 cells migrated in response to fMLP, CCL2/MCP-1, CXCL12/Stromal Cell-Derived Factor-1α (SDF-1α) and c-suPAR, but not to suPAR (Figure 5A). CCS of uninfected tonsil histocultures (Nil-c) failed to induce chemotaxis of U1 cells and removal of both suPAR forms did not affect this lack of response (Figure 5B, left panel). When CCS derived from either R5 or X4 infected tonsils (R5-c or X4-c) were tested for induction of U1 cell chemotaxis, no activity was detected; however, immunodepletion of both suPAR and c-suPAR from CCS of infected histocultures resulted in a significant migration of U1 cells. Of importance, addition of c-suPAR, but not of suPAR, to the immuno-depleted CCS inhibited the chemotactic response (Figure 5B, middle and right panels).

None of the above mentioned chemotactic stimuli tested induced HIV-1 expression in U1 cells (Figure 5C), whereas virus expression was detectable following PMA stimulation (Figure 5C). In contrast, CCS from uninfected tonsil (Nil-c) induced virus expression and removal of both suPAR and c-suPAR further increased it (Figure 5D). Of note is the fact that the reconstitution of the immunodepleted CCS with c-suPAR, but not with suPAR, decreased the levels of virus expression to that of non-immunodepleted supernatant (Figure 5D). In contrast, although also CCS from either R5 or X4 infected tonsils (R5-c or X4-c, respectively) induced virus expression from U1 cells, removal of both suPAR forms reduced the levels of virus expression (Figure 5D). Addition of c-suPAR, but not of suPAR, restored the levels of virus expression to that observed in non-immunodepleted supernatant (Figure 5D). Therefore, the contribution of c-suPAR to chemotaxis and HIV expression was opposite in the context of CCS obtained from infected and uninfected tonsils in that c-suPAR did not modulate chemotaxis but inhibited HIV expression induced by CCS from uninfected tonsils, whereas it inhibited the chemotactic potential and contributed to HIV-1 expression induced by CCS from infected tonsils. Although in the present study we have not further investigated the underlying mechanisms responsible for this functional dichotomy, it is conceivable that c-suPAR might interfere with the intracellular signaling pathways induced by different factors present in the CCS of infected and uninfected histocultures, known to secrete different levels of cytokines and chemokines [56], [57] able to influence both chemotaxis and HIV-1 expression.

### Expression of the PA System Elements in Tonsil Histocultures of a Single HIV-1^+^ Individual (MA35) Receiving cART

**Figure 6:**
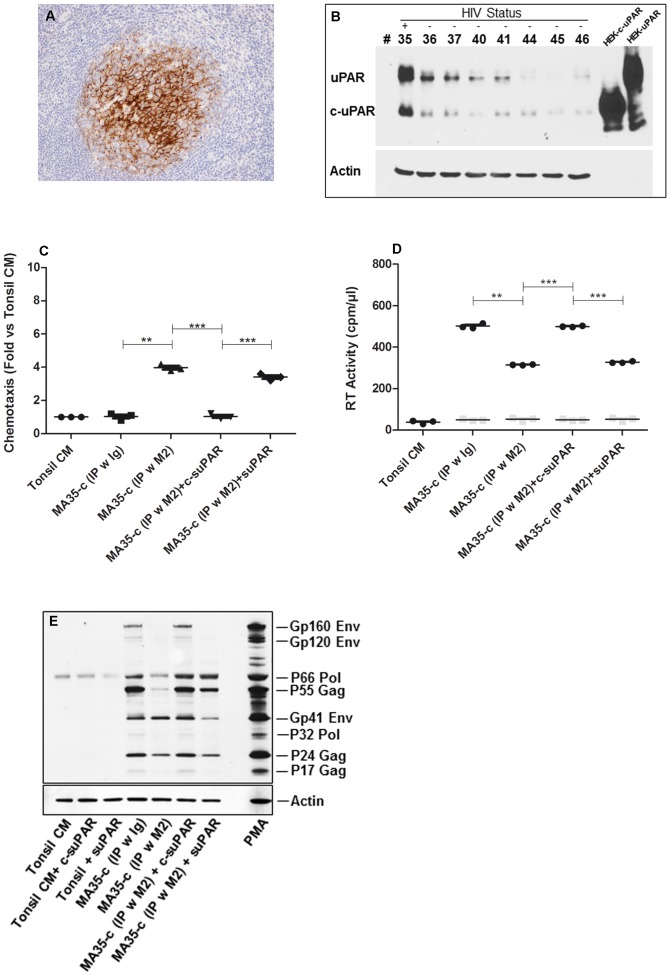
Plasminogen activator system in tonsils of HAART-treated HIV-1+ individual. Tonsils from one HIV-1+individual under cART (MA35) tested positive for HIV p24Gag in the germinative center of tonsil (panel A, OM 10x), and expressed both uPAR and c-uPAR (panel B; seven tonsils from uninfected individuals (#36–46) were used as reference control). Sections used for Western blotting were from snap-frozen tissue blocks, whereas lysates from stably transfected HEK cells expressing uPAR and c-uPAR were used as positive controls; because of high levels of expression of these antigens in HEK cells actin was not detected at exposure time used for showing actin from tissue lysate. Chemotaxis of (panel C) and HIV expression from (panel D) U1 cells were measured as described in Figure 3. Gray squares in panel D represent the value of input virus in the conditioned supernatants at the end of the 4 days of culture. Cell-associated HIV proteins were measured in U1 cells cultivated for 4 days with the stimuli reported at the bottom of the panel (E) and viral proteins were identified based on their molecular weight (right side of the panel). PMA (10 nM) was used as positive control for inducing virus expression (n = 3).

The hyperplastic tonsils of a single HIV-1^+^ individual receiving cART were collected, processed and cultured as above reported for tissues of uninfected individuals. Although cART induced undetectable levels of plasma viremia (<37 copies/ml for this individual at the time of tonsillectomy), the tonsils from this individual were positive for HIV p24_Gag_ expression (Figure 6A). In addition, MA35’s tonsils expressed uPAR and c-uPAR at higher levels than what observed in tonsils from uninfected individuals (Figure 6B). Higher level of suPAR were measured after 3 days of his tonsil histocultures vs. those established from 31 uninfected individuals (18,601 vs. 7,125±2461 pg/ml, respectively; n = 31, day 3 for Nil, Figure S1). Both full-length and c-suPAR forms were detected (Figure S3).

As observed with tonsil histocultures infected *ex vivo*, the CCS from MA35 revealed that c-suPAR, but not suPAR, inhibited U1 cell chemotaxis (Figure 6C). CCS from MA35 histoculture sustained HIV-1 expression from U1 cells, as evaluated both in the culture supernatant (Figure 6D) and at cellular level (Figure 6E) and the removal of both suPAR and c-suPAR from CCS by use of the M2 Ab resulted in decreased expression of HIV-1 proteins in U1 cells. Addition of c-suPAR (but not suPAR) to the immunodepleted MA35’s CCS restored normal levels of RT activity released in the culture supernatant (Figure 6D) and expression of viral proteins (Figure 6E). Thus, we can hypothesize that the pathway(s) inducing virus expression in the presence of c-suPAR is likely acting at the transcriptional level, at least in U1 cells. Further studies will assess the mechanism sustained by c-suPAR inducing HIV transcription, either by c-suPAR inducing intracellular signaling or by its ability to modulate signalings by different ligands.

## Discussion

In the present study, we have observed that HIV-1 infection of secondary lymphoid organ histocultures induced an increase in the number of macrophages, FDC and endothelial cells expressing uPAR. However, such an increase was observed only in hyperplastic lymphoid organs, and not those with normal histology, of HIV-1^+^ individuals suggesting an indirect role for infection in terms of promoting a state of immune activation. In addition, we have observed that HIV-1 infection of lymphoid histocultures enhanced the expression of cell-associated uPAR and c-uPAR and their release in culture supernatants, independently of the levels of virus replication as it was also observed when the infection was carried on in the presence of Lamivudine. These observations were confirmed in tonsil histocultures established from a single HIV-1 infected individual receiving cART. Finally, we collected evidence that c-suPAR, but not full length suPAR, present in CCS interferes with both chemotaxis and HIV-1 expression in U1 cells although with an opposite pattern as a function of whether the CCS were derived from infected or uninfected histocultures.

Our findings extend the characterization of the PA system to the pathophysiology of secondary lymphoid organs, relevant sites of viral persistency even under cART, and identify lymphoid organs as one of the main site of production and release of soluble forms of uPAR. In particular, we suggest that upregulation of suPAR expression is not directly linked to the levels of virus replication in lymphoid organs, but it is more likely mediated by modification of the extracellular microenvironment consequent to virus infection. In this regard, our findings support previous reports showing that a state of chronic inflammation caused increased levels of cell-associated uPAR on several cell types, including HIV-target cells and endothelial cells [58], [59], [60] and that the levels of suPAR in HIV-1^+^ individuals under cART were correlated to those of soluble TNF receptors [61].

The increased levels of suPAR detected in *ex vivo* infected tonsil histocultures were ca. 50% higher than those of parallel uninfected tissues. Of note is the fact that the same range of suPAR increase has been correlated with progression and poor prognosis in different diseases [62], [63], including HIV-1 infection [20] and cancer [64], sharing some levels of systemic inflammation and chronic immune activation as common denominator. Moreover, lymphoid organs released both suPAR and c-suPAR, indicating that they are a source of plasma-associated suPAR and c-suPAR, likely contributing to the immune dysfunction and systemic diseases typically observed in HIV-infected individuals. For example, binding of suPAR to ß3 integrin expressed by podocytes was correlated to the pathogenesis of focal segmental glomerulosclerosis [65], a disease also associated to HIV-1 infection [66].

We and others have previously investigated the uPA and HIV-1 infection in primary cells or cell lines. In these systems we reported that uPA inhibited the replication or expression of HIV-1 whereas suPAR prevented its effects [32]. However, the potential role of the cleaved form of the receptor was not investigated in these cells. We here report that c-suPAR modulated monocyte chemotaxis and HIV expression induced by the CCS of both uninfected and HIV-1 infected tonsil histocultures. The increased level of c-suPAR was responsible for blocking the migration of U1 cells and boosting HIV-1 expression by U1 cells in response to factor(s) present in CCS from *ex-vivo* infected histocultures and histocultures established from a single infected individual receiving cART.

In spite of the high levels of inflammatory cytokines during chronic HIV disease [67] there is no inflammatory infiltrate in tissues and organs, differently from what is observed with bacterial infections in which the PA system plays a defensive mechanism by favoring recruitment of neutrophils and monocytes [68]. Because in both bacterial and HIV infections the levels of both suPAR and c-suPAR are increased, these findings suggest that c-suPAR might participate to the different immune defense strategies. Indeed, activities of c-suPAR are different in the context of uninfected tonsils, unveiling its ability to influence biological processes in response to pathological events as consequent to the modification of the extracellular environment, as here tested in terms of CCS.

In this study we did not assess for the different mechanism responsible for the dichotomous effect of c-suPAR in the context of uninfected and HIV-1 infected lymphoid organs. However, the increased levels of CCL2 and c-suPAR observed in HIV-1 infected tonsils (irrespectively of the viral strain) might suggest that a common mechanism through which the chemotactic signal triggered by two ligands leads to cross-interference, as previously reported for CCL2 and CCL5 [69], whereas removal of c-suPAR unleashes the chemotactic potential of CCL2.

As to virus expression, both cytokines and chemokines over-expressed by HIV infected tonsils [56], [57], including CCL2 as shown in this study, own the capacity of modulating HIV-1 replication [67]. In this regard, it is of particular note that the intracellular signaling by FPRL1, the receptor engaged by c-suPAR, has been previously reported to mediate heterologous desensitization of CCR5 and CXCR4, thus inhibiting cellular entry of CCR5- and CXCR4-using HIV strains [70]. Nonetheless, FPRL1 can also be engaged by some HIV envelopes to gain entry into target cells [71], [72]. Among HIV target cells, FPRL1 is expressed by monocytes and macrophages, and chemokines such as CCL5/RANTES [73] and CCL3/MIP-1α [74] sustain virus replication and inhibit HIV post-entry steps in macrophages, respectively. Thus, engagement of FPRL1 by c-suPAR can profoundly modulate HIV-1 replication. Further studies directed toward the characterization of pathways modulated by c-suPAR are necessary to clarify the role of the PA system in HIV-1 pathogenesis, in particular addressing for the role of infected macrophages in lymphoid organs. In this regard, pprevious reports have investigated the uPA and HIV infection in primary cells or cell lines, thus lacking the proper environment, structure and complexity typical of lymphoid organs. Nonetheless, the action mechanism of the cleaved form of the receptor, such as modulation of biological activities by tissue-derived extracellular environment, could not have been properly revealed with models using single cell population or *in vitro* cell stimulation/activation/differentiation. Therefore, a novel aspect of this study is the definition of the distinct roles of the two forms of suPAR in the tonsil-derived extracellular environment.

Thus, we here provide evidence that the uPA system in lymphoid organs is affected by HIV-1 infection, resulting in increased expression and release of both the suPAR and c-suPAR forms. In addition, we provide evidence that c-suPAR might play a regulatory role in HIV-1 infection of secondary lymphoid organs, by interfering with both chemotaxis and virus replication. Our findings also provide a framework for further studies aiming to assess if suPAR represents a feed-forward loop sustaining virus expression while influencing the presence of HIV-target cells in lymphoid organs of HIV-1^+^ individuals even in the presence of cART (in which condition suPAR levels remain elevated).

In conclusion, our study identifies the cleaved form of suPAR as a candidate therapeutic target to be considered in cART-treated individuals for its potential to modulate the state of immune activation and residual viremia, as well as in the context of protocols aiming at reactivating latent provirus to achieve a functional cure of HIV-1 infection [75].

## Supporting Information

Figure S1 **Absolute levels of HIV replication, number of dead cells, uPA, PAI-I, suPAR and MCP-1 in culture supernatants**. Levels of reverse transcriptase activity (indicative of productive virus replication), number of death cells (estimated by the levels of LDH), and of CCL2/MCP-1, uPA, PAI-1 and suPAR were measured in culture supernatants collected every 3 days post infection. Vertical and horizontal bars represent mean and standard error of the mean. **(A)** Two-tailed paired T test was used to analysis efficiency of infection at each time point of culture. The absolute levels of replication over the uninfected tissue block reached statistical significance at day 6, day 9 and day 12 (p<0.0001 for both R5 and X4 strain vs. Nil, n = 31), and not statistical significance between R5 and X4 strains (p = 0.28 at day 6, p = 0.16 at day 9, p = 0.48 at day 12, n = 31) (Figure 1A). **(B)** To estimate the number of dead cells soluble levels of lactate dehydrogenase (LDH) [46] were measured in culture supernatants collected every 3 days post infection, and converted into number of dead cells by using standard curve prepared from known numbers of PBMC that underwent 5 cycles of freez-thaw [32]. Absolute numbers of dead cells were not different between Nil vs. R5 infected tissue blocks, Nil vs. X4 infected tissue blocks and R5 vs. X4 infected tissue blocks (Figure 2B), with data analyzed by two-tailed paired T test (n = 30). **(C)** Levels of CCL2/MCP-1 were evident during the entire period of histoculture, and the two-tailed paired T test revealed statistical difference between uninfected and R5 or X4 infected tissue blocks at day 9 and 12 of culture (p<0.0001 for R5 vs. Nil, p = 0.0024 for X4 vs. Nil at day 9; p<0.0001 for both R5 and X4 strain vs. Nil at day 12, n = 28). No statistical significance between R5 and X4 strains was monitored at each time point (p = 0.58 at day 9, p = 0.13 at day 12, n = 28). Indeed, statistical analysis (two-tailed paired t test) revealed that fold of CCL2/MCP-1 levels from HIV infected tissue blocks increased from day 6 to day 9 (R5 day 6 vs. day 9 p<0.0001; X4 day 6 vs. day 9 p = 0.0009, n = 26) reaching a plateau at day 12 (R5 day 9 vs. day 12 p = 0.42; X4 day 9 vs. day 12 p = 0.95, n = 26), along the increased virus replication. **(D)** Absolute levels of suPAR increased during the entire period of histoculture, and statistical significance between uninfected and HIV infected tissue blocks was reached at day 12 post infection (two-tailed paired t test, Nil vs. R5 infected tissue blocks p = 0.0023, Nil vs. X4 infected tissue blocks p = 0.0061, n = 28). No statistical significance between R5 and X4 strains was monitored at each time point, including day 12 post-infection (p = 0.93, n = 28). SuPAR levels from HIV infected tissue blocks increased from day 6 to day 9 (R5, day 6 vs. day 9 p<0.0001; X4, day 6 vs. day 9 p<0.0001, n = 26) reaching a plateau at day 12 (R5, day 9 vs. day 12 p = 0.27; X4, day 9 vs. day 12 p = 0.84, n = 28). **(E)** Absolute levels of uPA increased over the entire period of culture, in particular after six days of culture (two-tailed paired t test; Nil at day 6 vs. Nil at day 9 p = 0.0022, n = 26), and both R5 and X4 infection did not modify its expression when compared to uninfected tissue blocks. **(F)** Absolute levels of PAI-1 increased after 3 days of culture and then remained constant up to day 12 of culture, and both R5 and X4 infection did not modify its expression.
(TIF)

Figure S2 **In vitro HIV infection and modulation of the number of HIV+ and uPA+ cells.** IHC analysis for HIV p24Gag and uPA antigens was used for estimating the number of cells expressing virus and uPA at day 0, 6 and 12 post infection. **(A)** HIV p24+ cells were detected starting from 6 days of histoculture. No difference between viral strains was observed. **(B)** UPA+ cells were present in tonsils at the time of surgical removal (Day 0), increased during the first six days of culture (day 0 vs. day 6 p<0.0001, n = 7) and remained constant in the following week. Both R5 and X4 infection did not modify the number of uPA+ cells over than what observed in uninfected tissue blocks. Data were analyzed by two-tailed paired T test, and p values indicated by asterisks (** = 0.001–0.01; *** = <0.001).
(TIF)

Figure S3 **Tonsils of HAART-treated HIV+ individual released both full-length and cleaved suPAR.** UPAR forms and HIV p24Gag were evaluated in the conditioned supernatant after 3 days of histoculture of tonsils from MA35. In parallel, conditioned supernatants from in vitro HIV infected tonsils were also measured.
(TIF)
