# Supplementary figures and images for: HIV-1 Infected Lymphoid Organs Upregulate Expression and Release of the Cleaved Form of uPAR That Modulates Chemotaxis and Virus Expression
Source: PLoS One. 2013 Jul 29;8(7):e70606. doi: 10.1371/journal.pone.0070606 (PMC3726662; doi:10.1371/journal.pone.0070606)

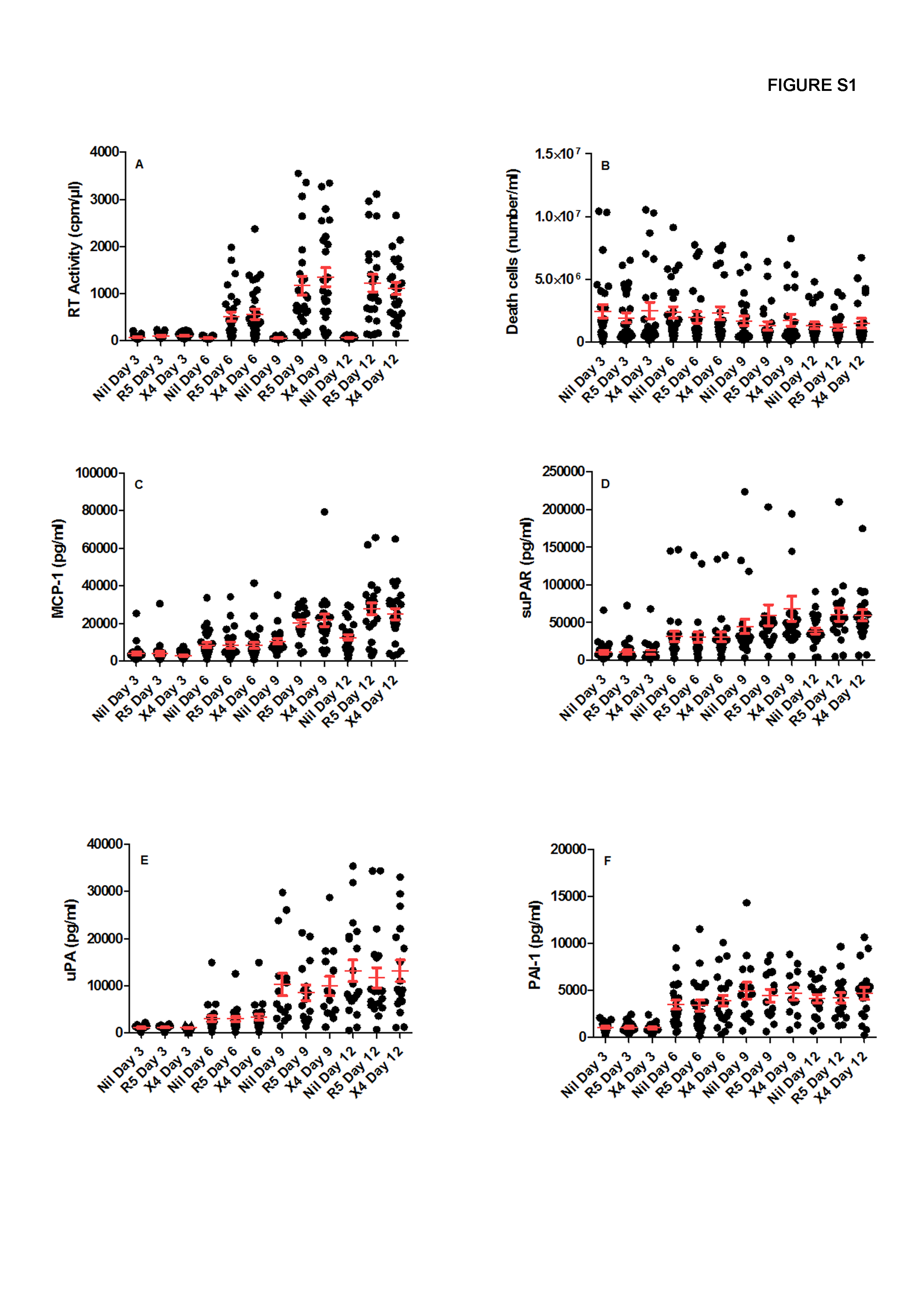

Supplement: Figure S1 — Absolute levels of HIV replication, number of dead cells, uPA, PAI-I, suPAR and MCP-1 in culture supernatants. Levels of reverse transcriptase activity (indicative of productive virus replication), number of death cells (estimated by the levels of LDH), and of CCL2/MCP-1, uPA, PAI-1 and suPAR were measured in culture supernatants collected every 3 days post infection. Vertical and horizontal bars represent mean and standard error of the mean. (A) Two-tailed paired T test was used to analysis efficiency of infection at each time point of culture. The absolute levels of replication over the uninfected tissue block reached statistical significance at day 6, day 9 and day 12 (p<0.0001 for both R5 and X4 strain vs. Nil, n = 31), and not statistical significance between R5 and X4 strains (p = 0.28 at day 6, p = 0.16 at day 9, p = 0.48 at day 12, n = 31) (Figure 1A). (B) To estimate the number of dead cells soluble levels of lactate dehydrogenase (LDH) [46] were measured in culture supernatants collected every 3 days post infection, and converted into number of dead cells by using standard curve prepared from known numbers of PBMC that underwent 5 cycles of freez-thaw [32]. Absolute numbers of dead cells were not different between Nil vs. R5 infected tissue blocks, Nil vs. X4 infected tissue blocks and R5 vs. X4 infected tissue blocks (Figure 2B), with data analyzed by two-tailed paired T test (n = 30). (C) Levels of CCL2/MCP-1 were evident during the entire period of histoculture, and the two-tailed paired T test revealed statistical difference between uninfected and R5 or X4 infected tissue blocks at day 9 and 12 of culture (p<0.0001 for R5 vs. Nil, p = 0.0024 for X4 vs. Nil at day 9; p<0.0001 for both R5 and X4 strain vs. Nil at day 12, n = 28). No statistical significance between R5 and X4 strains was monitored at each time point (p = 0.58 at day 9, p = 0.13 at day 12, n = 28). Indeed, statistical analysis (two-tailed paired t test) revealed that fold of CCL2/MCP-1 lev [file pone.0070606.s001.tif]

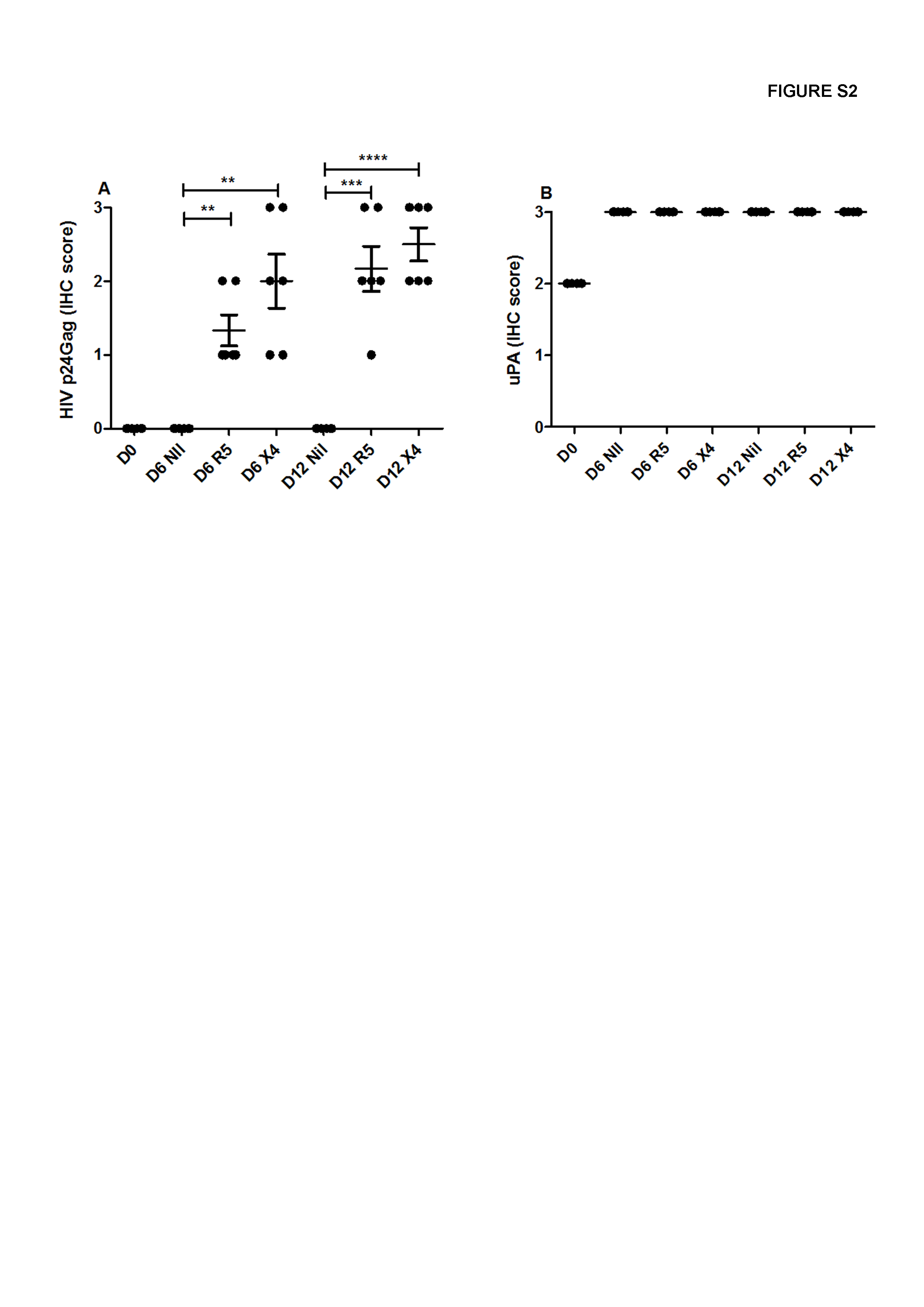

Supplement: Figure S2 — In vitro HIV infection and modulation of the number of HIV+ and uPA+ cells. IHC analysis for HIV p24Gag and uPA antigens was used for estimating the number of cells expressing virus and uPA at day 0, 6 and 12 post infection. (A) HIV p24+ cells were detected starting from 6 days of histoculture. No difference between viral strains was observed. (B) UPA+ cells were present in tonsils at the time of surgical removal (Day 0), increased during the first six days of culture (day 0 vs. day 6 p<0.0001, n = 7) and remained constant in the following week. Both R5 and X4 infection did not modify the number of uPA+ cells over than what observed in uninfected tissue blocks. Data were analyzed by two-tailed paired T test, and p values indicated by asterisks (** = 0.001–0.01; *** = <0.001). (TIF) [file pone.0070606.s002.tif]

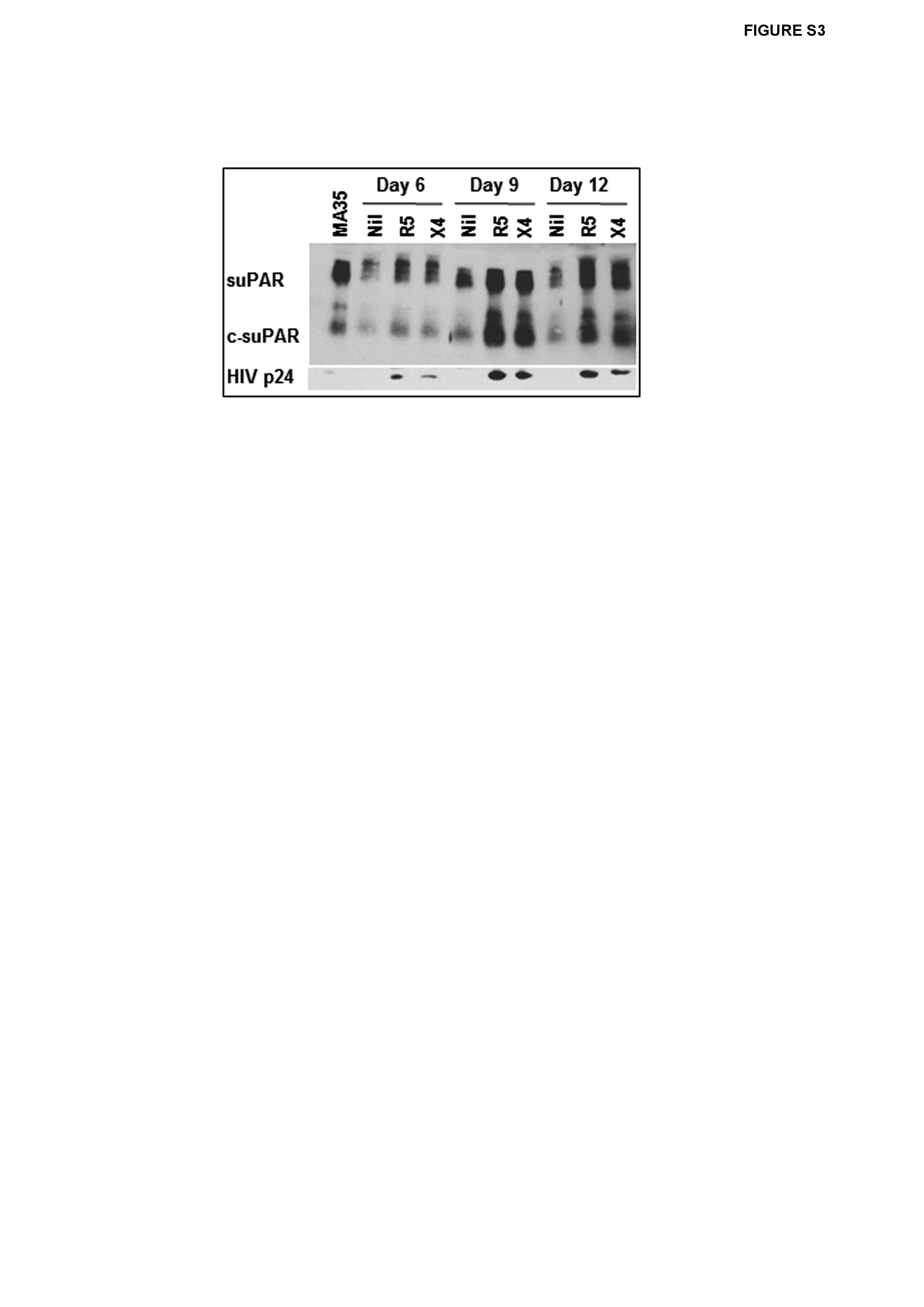

Supplement: Figure S3 — Tonsils of HAART-treated HIV+ individual released both full-length and cleaved suPAR. UPAR forms and HIV p24Gag were evaluated in the conditioned supernatant after 3 days of histoculture of tonsils from MA35. In parallel, conditioned supernatants from in vitro HIV infected tonsils were also measured. (TIF) [file pone.0070606.s003.tif]
